# Establishment of MDCK Stable Cell Lines Expressing TMPRSS2 and MSPL and Their Applications in Propagating Influenza Vaccine Viruses in Absence of Exogenous Trypsin

**DOI:** 10.1155/2015/402628

**Published:** 2015-03-30

**Authors:** Zhiyuan Wen, Chao Wu, Weiye Chen, Xianying Zeng, Jianzhong Shi, Jinying Ge, Hualan Chen, Zhigao Bu

**Affiliations:** State Key Laboratory of Veterinary Biotechnology, Harbin Veterinary Research Institute, Chinese Academy of Agricultural Sciences, 427 Maduan Street, Harbin 150001, China

## Abstract

We established two Madin-Darby canine kidney (MDCK) cell lines stably expressing human airway transmembrane protease: transmembrane protease, serine 2 (TMPRSS2) and mosaic serine protease large form (MSPL) which support multicycle growth of two H5 highly pathogenic avian influenza viruses (HPAIV) recombinant vaccines (Re-5 and Re-6) and an H9 avian influenza virus (AIV) recombinant vaccine (Re-9) in the absence of trypsin. Data showed that the cell lines stably expressed TMPRSS2 and MSPL after 20 serial passages. Both MDCK-TMPRSS2 and MDCK-MSPL could proteolytically cleave the HA of Re-5, Re-6, and Re-9 and supported high-titer growth of the vaccine without exogenous trypsin. Re-5, Re-6, and Re-9 efficiently infected and replicated within MDCK-TMPRSS2 and MDCK-MSPL cells and viral titer were comparable to the virus grown in MDCK cells with TPCK-trypsin. Thus, our results indicate a potential application for these cell lines in cell-based influenza vaccine production and may serve as a useful tool for HA proteolytic cleavage-related studies.

## 1. Introduction

Influenza is a major zoonotic threat to public health, which is caused by 3 types (A, B, and C) of influenza viruses [1, 2]. Type A influenza is the most serious type, specifically the highly pathogenic H5N1 [3–5], H1N1 [5–7], and the newly emerged lethal H7N9 [8, 9].

Hemagglutinin (HA) of influenza virus mediates both receptor binding and membrane fusion [10]. HA cleavage is important for viral infectivity; HA proteins are synthesized as HA0 precursor proteins during transport through the Golgi apparatus. HA0 is cleaved by host cell protease into HA1 and HA2 subunits [11, 12]. Cleaved HA proteins bind to cell receptor and then are endocytosed into the endosome where they undergo conformational changes and exposure of fusion peptide on HA2 subunit under low pH. Then, the fusion peptide is inserted into the cell membrane and mediates the formation of fusion pore [13, 14]. Fusion relies on precise HA0 cleavage for a fusion-capable HA2 subunit. HA proteins of H5 highly pathogenic avian influenza viruses (HPAIV) have multibasic cleavage sites (R-X-R/K-R) which can be cleaved by ubiquitously expressed furin or PC5/6 protease to cause fatal systemic infections [15–17]. HA of most of the other mammalian and avian influenza viruses contains a single arginine (or lysine) at the cleavage site, so cleavage of these HAs is restricted to the respiratory tract in mammals and to the respiratory and intestinal tracts in avians and assumed to be processed extracellularly by trypsin-like proteases. Of these proteases, some type II transmembrane serine proteases (TTSPs) family members such as human airway trypsin-like (HAT) protease, transmembrane protease, serine 2 (TMPRSS2), transmembrane protease, serine 4 (TMPRSS4), and mosaic serine protease large form (MSPL) play important roles in influenza viral infection. TTSPs are expressed in the airways and can cleave multiple strains of influenza HA protein. Böttcher and colleagues reported a cell-associated cleavage of influenza viruses HA with a monobasic cleavage site by HAT and TMPRSS2 [18]. Wang and colleagues reported that TMPRSS2 and HAT could cleave the HA of the H1, H3, and H5 subtypes [19]. Zmora and colleagues demonstrated that mosaic serine protease large form (MSPL)could activate HA protein of H1N1 and H3N2 influenza virus [20], while Okumura and colleagues confirmed that MSPL can cleave the HA protein of H5 HPAIV and support their multicycle replication [21].

Here, we established two MDCK cell lines that stably express TMPRSS2 and MSPL. Western blot and RT-PCR confirmed the presence of the target gene; FACS assay confirmed target gene expression in serially passaged cells. Cell fusion assay indicated that TMPRSS2 and MSPL cell lines could cleave the HA protein of H5 and H9 subtypes. Both cell lines can support multicycle growth of Re-5, Re-6, and Re-9 in absence of exogenous trypsin. Vaccine titers of these cell lines were comparable to those in MDCK cells plus TPCK-trypsin.

## 2. Materials and Methods

### 2.1. Viruses and Cells

Low-passage Madin-Darby canine kidney (MDCK) cells were maintained in DMEM containing 10% fetal bovine serum (FBS). Influenza viruses Re-5 [22], Re-6 [23], and Re-9 were provided by the National Animal Influenza Reference Laboratory. Viruses were generated with a “6 + 2” strategy: all three viruses contained 6 internal genes from A/Puerto Rico/8/1934 (H1N1).* HA* and* NA* genes of Re-5 were from A/Duck/Anhui/1/2005 (H5N1);* HA* and* NA* genes of Re-6 were from A/Duck/Guangdong/s1322/2010 (H5N1); and* HA* and* NA* genes of Re-9 were from A/Chicken/Hunan/S933/2008 (H9N2). To enhance safety, the multibasic amino acid cleavage site of the HA protein of Re-5 (RRRRKR) and Re-6 (RERRRKR) was changed to monobasic amino acids (RETR).

### 2.2. Generation of MDCK-TMPRSS2 and MDCK-MSPL Stable Cell Lines

Human* TMPRSS2* (GenBank number U75329.1) and human* MSPL* (GenBank number AB048796.1) genes were synthesized by Generay Biotech (Shanghai, China), and both genes were fused to a Flag tag (DYKDDDDK) at the 3′-end of the ORF. Eukaryotic expression vector P4 was used to obtain the stable cell lines. P4 was derived from the pCAGGS vector and this was modified by inserting an enhanced* GFP* (eGFP) gene and G418-resistant gene (*neo*) to render it suitable for establishing a stable cell line. Either TMPRSS2-FLAG or MSPL-FLAG was inserted into the P4 vector multiple cloning site which is located upstream of the IRES and eGFP genes, allowing the inserted genes to be detected with FLAG and green fluorescence. The resultant plasmids, P4-TMPRSS2-FLAG and P4-MSPL-FLAG, were prepared and purified by Qiagen Maxi Prep plasmid kit (Qiagen, Valencia, CA) before transfection.

Newly recovered, low-passage MDCK cells were serially passaged 5 times. Cells were then electrotransfected following the protocol of Nucleofector Kit for MDCK cells (Lonza, Cologne, Germany). Briefly, 5 × 10^5^ MDCK cells were resuspended in 0.1 mL Solution I (provided by the kit). 5 *μ*g plasmid (P4-TMPRSS2-FLAG or P4-MSPL-FLAG) was added to Solution I within 15 min. The cell-plasmid mix was transferred to a specialized cuvette which was then loaded to an Amaxa Nucleofector II machine (Lonza, Cologne, Germany) for electrotransfection using the A24 program. After electrotransfection, cells were transferred to 6-well plates containing DMEM with 10% FBS. 48 h later, attached monolayer cells were trypsinized, diluted 1 : 100, and seeded into 24-well plates containing DMEM with 5% FBS and 800 *μ*g/mL G418. Medium was replaced every 3 days and cultured for 2 weeks after which G418-resistant cell colonies were collected and transferred to new plates. Cells were then trypsinized and loaded to a BD FACSAria cell sorter; single green fluorescence positive cells were sorted to 96-well plates and cultured under G418 pressure. Two weeks later, surviving cell colonies were transferred to 48-well plates and continued to be cultured in presence of G418. Cells were then scaled up and cultured for at least 10 passages to generate MDCK-TMPRSS2 and MDCK-MSPL cells.

### 2.3. Verification of TMPRSS2 and MSPL Expression in Cell Lines

For RT-PCR, total RNA of 5 × 10^6^ MDCK-TMPRSS2 or MDCK-MSPL cells was extracted with a Qiagen RNAeasy Mini Kit (Qiagen, Valencia, CA). RNA was reverse transcribed with a Promega Improm II RT kit (Promega, Madison, WI). The following primers were used to amplify TMPRSS2 or MSPL with PrimeStar HS polymerase (Takara, Dalian, China): TMPRSS2-F 5′-ATGGCTTTGAACTCAG-3′ and TMPRSS2-R 5′-TTACTTATCGTCGTCATC-3′; MSPL-F: 5′-ATGGAGAGGGACAGCCAC-3′ and MSPL-R 5′-TTACTTATCGTCGTCATC-3′.

For Western blot, MDCK-TMPRSS2, MDCK-MSPL, or MDCK cells were washed with PBS, lysed with RIPA buffer (containing 25 mM Tris-HCl pH 7.6, 150 mM NaCl, 1% NP-40, 1% desoxycholic acid sodium salt, and 0.1% SDS) (Thermo Fisher, Waltham, MA), and mixed with reducing protein loading buffer. Samples were separated with SDS-PAGE and subsequently transferred to a nitrocellulose (NC) membrane (GE Healthcare, Pittsburgh, PA). NC membranes were incubated with 1 : 100 diluted mouse anti-FLAG antibody (Abcam, Cambridge, MA) as primary antibody and 1 : 4000 diluted HRP labeled goat anti-mouse IgG (Sigma, St. Louis, MO) as secondary antibody. NC membranes were then incubated with ECL plus reagent (GE Healthcare, Pittsburgh, PA) and protein bands were visualized on a Kodak XAR films (Kodak).

For FACS assay, 2 × 10^6^ of the 10th serially passaged MDCK-TMPRSS2 and MDCK-MSPL cells were collected and washed with PBS and then filtered through a 40-*μ*m cell strainer (BD Biosciences, San Jose, CA). Cells were then loaded to a BD FACSAria machine to measure green fluorescence. Data were analyzed with FlowJo (Treestar Inc., Ashland, OR) software and expressed as histogram.

### 2.4. Cell Fusion Assay

MDCK cells were seeded in 6-well plates, and 1 *μ*g of pCAGGS-TMPRSS2-FLAG, pCAGGS-MSPL-FLAG, and pCAGGS was cotransfected with 1 *μ*g of pCAGGS-Re-5HA, pCAGGS-Re-6HA, and pCAGGS-Re-9HA, respectively, using Lipofectamine 3000 reagent (Invitrogen, Carlsbad, CA). 48 h after transfection, cells were treated with acidic PBS (pH 5.0) at room temperature for 5 min and replenished with DMEM containing 5% FBS and cultured at 37°C for 1 h. When fusion was obvious, cells were fixed with 3% paraformaldehyde and stained with mouse anti-FLAG antibody and chicken anti-Re-5, anti-Re-6, or anti-Re-9 as primary antibody with FITC-labeled goat anti-mouse antibody (Abcam, Cambridge, CA) and TRITC-labeled rabbit anti-chicken antibody (Abcam, Cambridge, CA) for 30 min. Cells were washed and stained with DAPI and images were acquired with a Leica TCS SP5 confocal laser microscope (Leica Microsystems, Wetzlar, Germany).

### 2.5. Immunofluorescence Assay

MDCK-TMPRSS2, MDCK-MSPL, and MDCK cells were seeded in 12-well plates and grown in DMEM containing 5% FBS for 24 h. Cells were infected with Re-5, Re-6, and Re-9 at MOI = 0.01 for 1 h at 37°C. Inoculum was removed and cells were washed 3 times and placed in DMEM with 5% FBS. 36 h after incubation, cells were washed with PBS and fixed with 3% paraformaldehyde at room temperature for 15 min. Infected cells were detected with chicken anti-Re-5, anti-Re-6, and anti-Re-9 as primary antibodies and TRITC-labeled goat anti-chicken IgG (Sigma, St. Louis, MO) as secondary antibody. Stained cells were observed under a Zeiss Axioscop (Zeiss, Thornwood, NY) fluorescent microscope.

### 2.6. Viral Titration

MDCK-TMPRSS2, MDCK-MSPL, and MDCK cells were seeded in 6-well plates and grown in DMEM containing 5% FBS for 24 h. Cells were infected with Re-5, Re-6, and Re-9 at MOI = 0.01 in DMEM for 1 h at 37°C. Inoculum was removed and cells were washed 3 times after which DMEM with 5% FBS was added. TPCK-treated trypsin (1 *μ*g/mL) was added to media of infected MDCK cells. 100 *μ*L supernatant from each infected cell culture was sampled at 12, 24, 48, 72, and 96 h after infection. Sample TCID_50_ values were determined, and growth kinetics for each virus from each cell line were obtained. Statistical analysis (*t*-test) was carried out by Excel software (Microsoft, Redmond, WA) for each virus between MDCK-TMPRSS2, MDCK-MSPL, MDCK + TPCK, and MDCK infection groups at the same time point.

## 3. Results

### 3.1. Stable Expression of TMPRSS2 and MSPL in MDCK Cells

**Figure 1:**
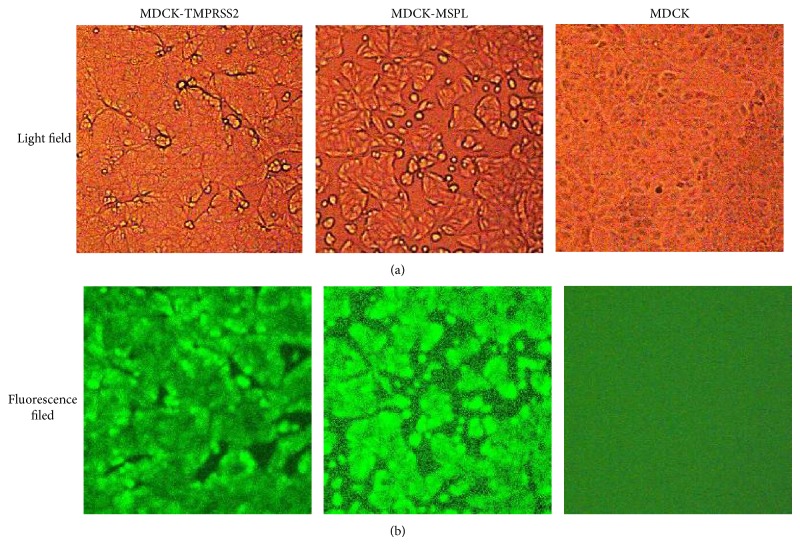
Morphology of MDCK-TMPRSS2 and MDCK-MSPL cells. MDCK-TMPRSS2, MDCK-MSPL, and MDCK cells were observed under a fluorescent microscope. (a) Cells under a light field; (b) cells under a fluorescent field.

MDCK-TMPRSS2 and MDCK-MSPL cells were established by electrotransfection of plasmids encoding either TMPRSS2 or MSPL. After transfection, cells were serially passaged in presence of 800 *μ*g/mL G418. Surviving cells were cultured and scaled up for experiments. To detect TMPRSS2 and MSPL expression, a FLAG tag was in-frame fused to the C-terminus of the TMPRSS2 or MSPL. An eGFP gene was placed upstream of the FLAG-tagged TMPRSS2 or MSPL, which were linked by an IRES sequence. Thus TMPRSS2 or MSPL expression could be detected with FLAG antibody and green fluorescence. As shown in Figure 1, the morphology of TMPRSS2-MDCK and MSPL-MDCK cells was slightly different from MDCK cells. Under a fluorescent microscope, almost all TMPRSS2-MDCK and MSPL-MDCK cells were positive for green fluorescence except for MDCK cells.

**Figure 2:**
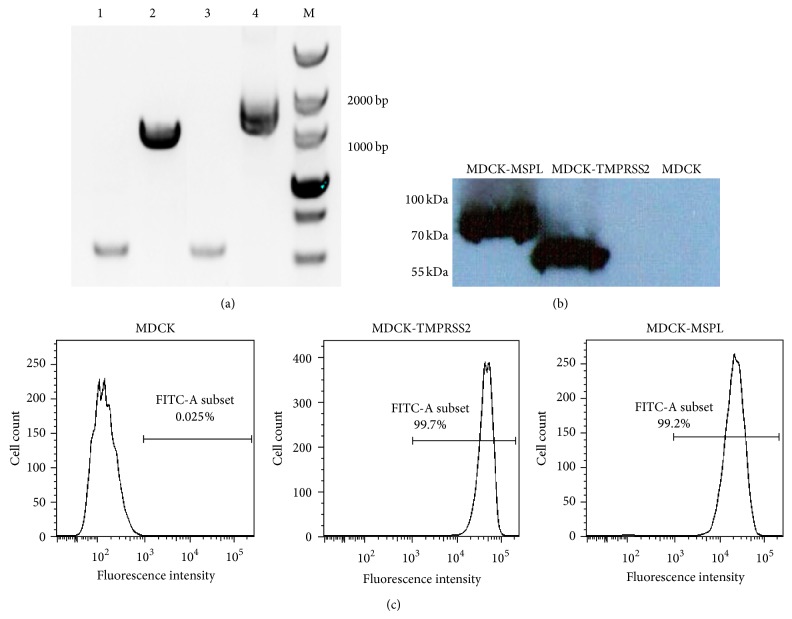
Stable expression of TMPRSS2 and MSPL in cell lines. (a) RT-PCR of MDCK-TMPRSS2 and MDCK-MSPL cells. Lanes 1 and 3: beta-actin; Lane 2: TMPRSS2; Lane 4: MSPL. (b) Western blot of MDCK-TMPRSS2 and MDCK-MSPL cells. Cell lysates were separated on SDS-PAGE and transferred to nitrocellulose membranes; TMPRSS2-FLAG and MSPL-FLAG fusion proteins were detected with FLAG antibody. (c) Green fluorescence of MDCK-TMPRSS2 and MDCK-MSPL cells was detected by flow cytometry. Both MDCK-TMPRSS2 and MDCK-MSPL cells were positive for green fluorescence.

RT-PCR was performed to test for the presence of* TMPRSS2* and* MSPL* genes in cell lines.  Figure 2 depicts a specific band amplified from total RNA of MDCK-TMPRSS2 or MDCK-MSPL cells and this confirmed the presence of* TMPRSS2* and* MSPL* genes. To measure expression of TMPRSS2 and MSPL, Western blot and flow cytometry were performed. As shown in Figure 2(b), a 70 kDa band indicated MSPL, and a 55 kDa band indicated TMPRSS2 and no band was detected in MDCK cells. As shown in Figure 2(c), flow cytometry revealed over 99% of TMPRSS2-MDCK and MSPL-MDCK cells to be positive for green fluorescence. Normal MDCK cells were negative. These data confirmed the stable expression of TMPRSS2 and MSPL by the cell lines.

### 3.2. Proteolytic Cleavage of HA Protein of Re-5, Re-6, and Re-9 by TMPRSS2 and MSPL

**Figure 3:**
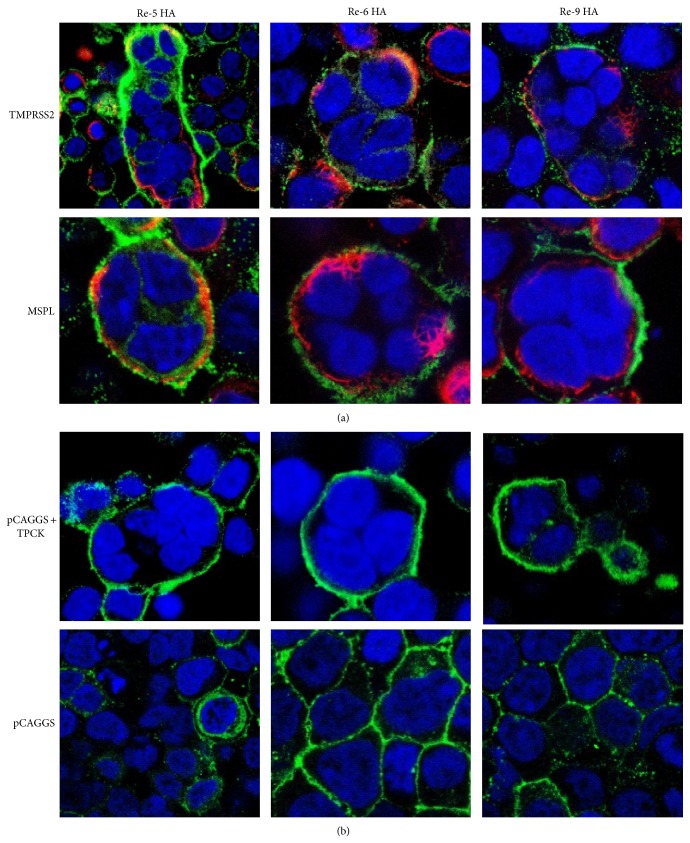
Cell fusion induced by cotransfection of protease and influenza HA under acidic conditions. MDCK cells were cotransfected with pCAGGS-TMPRSS2 plus pCAGGS-(Re-5, Re-6, and Re-9) HA and pCAGGS-MSPL plus pCAGGS-(Re-5, Re-6, and Re-9) HA, respectively (a). pCAGGS plus pCAGGS-(Re-5, Re-6, and Re-9) HA supplemented with TPCK-trypsin and pCAGGS plus pCAGGS-(Re-5, Re-6, and Re-9) HA without TPCK-trypsin as control groups (b). See methods for cell treatments. Stained cells were observed under a Leica confocal laser microscope.

To determine whether TMPRSS2 and MSPL could proteolytically activate the HA proteins of Re-5, Re-6, and Re-9, cotransfection and cell fusion assay were carried out. As shown in Figure 3, cell fusion was observed under a confocal microscope 48 h after transfection of TMPRSS2 + Re-5HA, TMPRSS2 + Re-6HA, and TMPRSS2 + Re-9HA, as well as MSPL + Re-5HA, MSPL + Re-6HA, and MSPL + Re-9HA. Cell fusion was also observed in the pCAGGS vector + HAs group in presence of TPCK-trypsin, whereas cell fusion failed in the pCAGGS vector + HAs group in absence of TPCK-trypsin. Fusion of HA relied on precise cleavage of HA and endosome acidification. These data showed that both TMPRSS2 and MSPL could proteolytically activate the HA protein of Re-5, Re-6, and Re-9.

### 3.3. Re-5, Re-6, and Re-9 Could Infect and Spread within MDCK-TMPRSS2 and MDCK-MSPL Cells in Absence of Exogenous Trypsin

**Figure 4:**
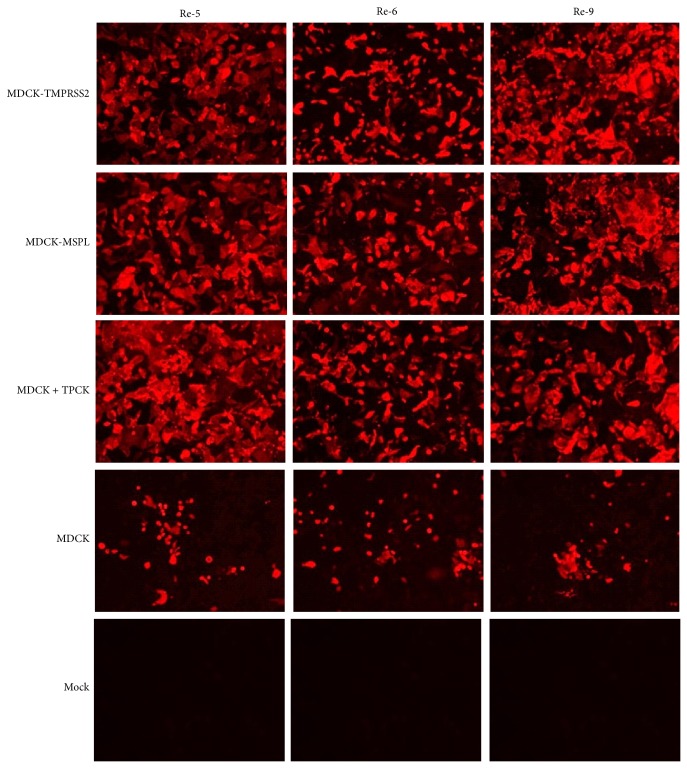
Re-5, Re-6, and Re-9 infected and spread within MDCK-TMPRSS2 and MDCK-MSPL cells. MDCK-TMPRSS2, MDCK-MSPL, MDCK + TPCK, and MDCK cells were infected with Re-5, Re-6, and Re-9, respectively (MOI = 0.01); cells were fixed 60 h after infection. Cells were incubated with Re-5, Re-6, and Re-9 antibodies and TRITC-labeled secondary antibody. Cells were observed under a Zeiss fluorescent microscope.

Re-5, Re-6, and Re-9 were used to infect MDCK-TMPRSS2 or MDCK-MSPL cells (MOI = 0.01). 60 h after infection, cells were washed and fixed, and 1 : 100 diluted chicken anti-Re-5, anti-Re-6, or anti-Re-9 serum was used as primary antibody. 1 : 200 diluted TRITC-labeled rabbit anti-chicken IgG was used as secondary antibody to detect the virus in infected cells. As shown in Figure 4, Re-5, Re-6, and Re-9 could infect and spread within MDCK-TMPRSS2 and MDCK-MSPL cells in absence of TPCK-trypsin, which was similar to MDCK cells with TPCK-trypsin. These results confirmed that MDCK-TMPRSS2 and MDCK-MSPL cells could proteolytically cleave HA protein of Re-5, Re-6, and Re-9 and support multicycle replication of these viruses in the cell lines.

### 3.4. MDCK-TMPRSS2 and MDCK-MSPL Cells Support High-Titer Growth of Re-5, Re-6, and Re-9 in Absence of Exogenous Trypsin

**Figure 5:**
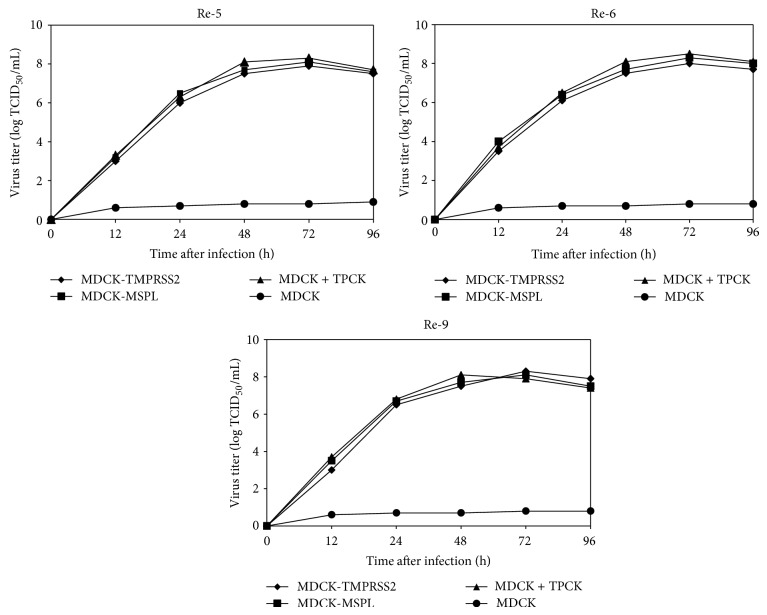
Growth kinetics of Re-5, Re-6, and Re-9 on MDCK-TMPRSS2 and MDCK-MSPL cells. MDCK-TMPRSS2, MDCK-MSPL, MDCK + TPCK, and MDCK cells were infected with Re-5, Re-6, and Re-9 as described and samples were taken at 12, 24, 48, 72, and 96 h after infection. Sample TCID_50_ were determined and calculated using the method of Reed and Muench.

Growth kinetics of Re-5, Re-6, and Re-9 on MDCK-TMPRSS2, MDCK-MSPL, and MDCK cells were measured. Growth curves of each virus in different cells were plotted.  Figure 5 depicts the growth kinetic data. Re-5, Re-6, and Re-9 in MDCK-TMPRSS2 and MDCK-MSPL cells were comparable to those with MDCK plus TPCK-trypsin (*P* > 0.05), while the viral titers in MDCK-TMPRSS2, MDCK-MSPL, and MDCK with TPCK-trypsin were significantly higher than those of MDCK cells without TPCK-trypsin (*P* < 0.01). Thus, MDCK-TMPRSS2 and MDCK-MSPL cells can well support high-titer growth of influenza viruses in absence of exogenous trypsin, suggesting a future application for industrial vaccine production.

## 4. Discussion

Cleavage of HA by host protease is the prerequisite for influenza viral infection. Most influenza viral HA proteins possess monobasic amino acid cleavage sites. Thus, HA must be proteolytically cleaved into HA1 and HA2 subunits to acquire cell fusion ability and infectivity. Many trypsin-like proteases which can cleave HA protein have been reported (plasmin, tryptase Clara, swine mast cell tryptase, chicken embryo trypsin-like protease, and coagulation factor Xa) [24, 25]. These proteases were isolated from tissues and biochemically characterized, but their molecular identity is unknown. Recently, human type II transmembrane serine proteases (TTSPs) have come under study and TTSPs such as HAT, TMPRSS2, TMPRSS4, and MSPL were found to be capable of cleaving different subtypes of influenza viruses. TMPRSS2 and HAT could cleave the HA protein of H1, H2, H3, and H5 viruses and the 1918 “Spanish flu” virus [18, 19, 26]. It is noteworthy that MSPL can activate different subtypes of influenza virus HA protein possessing either monobasic or multibasic amino acid cleavage site. Zmora and colleagues demonstrated that MSPL can activate the HA protein of H1 and H3 influenza virus as well as the 1918 “Spanish flu” virus [20]. While Kido and colleagues reported that MSPL can cleave an H5 influenza HA-derived multibasic peptide, which was not efficiently cleaved by furin [21, 27], the study of Okumura and colleagues confirmed that MSPL can activate the HA protein of H5 HPAIV and support their multicycle replication [21]. The versatile cleaving ability of MSPL is the major reason we use it to construct the stable cell line.

We have established two stable MDCK cell lines, MDCK-TMPRSS2 and MDCK-MSPL, which could proteolytically activate influenza virus HA and support multicycle growth of the influenza viruses. Re-5, Re-6, and Re-9 were used to produce inactivated commercial vaccines for China's bird flu control and infection. Using the “6 + 2” strategy, the 6 internal genes of PR8 and HA and the NA genes of other influenza viruses were used to generate recombinant viruses. Because PR8-based viruses can propagate to high titer easily in chicken eggs, they are suitable for making inactivated vaccines. To increase the safety of Re-5 and Re-6, the multibasic cleavage sequence was changed to monobasic sequence.

Cell-based vaccines are gaining popularity over egg-based vaccines because cell lines are well characterized and comply with regulatory guidelines. Also, cell culture media are chemically defined and yield consistent results. At present, two cell lines, MDCK cells and Vero cells, are approved and used for influenza vaccine production [28–31]. Cell-based influenza vaccines can provide equivalent protection in animals and humans compared to vaccines produced in chicken eggs [31–33] and MDCK cells have been recognized to be the most suitable cells for producing influenza vaccines [33–39]. Usually, exogenous trypsin is needed for cell-based vaccine production. However, we report that the virus propagated in MDCK-TMPRSS2 and MDCK-MSPL cells could reach an equivalent titer compared with the MDCK cells plus TPCK-trypsin. Thus, these cell lines can be used for cell-based vaccine production.

Böttcher and coworkers reported inducible MDCK cell lines which express TMPRSS2 and HAT under the control of tetracycline transcription system [40]. Their results showed that the cell lines can support multicycle replication of human H2 and H3 influenza viruses without trypsin. Different from their inducible cell lines, the cell lines we established consistently expressed TMPRSS2 or MSPL. Unexpectedly, permanent expression of these proteases was not toxic to the cells and both cell lines could be serially passaged for at least 20 passages. Even though these cells grew slightly slower than MDCK cells, when they grew to the suitable confluence for infection, the viral titer was comparable to the MDCK cells plus exogenous trypsin. Since Re-5, Re-6, and Re-9 are commercial inactivated AIV vaccines in China, we speculate that the cell lines could be applied in the large scale vaccine production in the future. They may also simplify the related researches or industrial applications to meet diverse demands.

Also, both MDCK-TMPRSS2 and MDCK-MSPL can cleave the HA protein of Re-5, Re-6, and Re-9 and support multicycle replication of these viruses. MDCK-TMPRSS2 and MDCK-MSPL cells could therefore be useful for studying cleavage activation of influenza virus HA by TMPRSS2 and MSPL. Finally, the cell lines may allow development of TMPRSS2 and MSPL-specific protease inhibitors.
